# Supplementary material for: Extracellular Vesicles from Human Advanced-Stage Prostate Cancer Cells Modify the Inflammatory Response of Microenvironment-Residing Cells
Source: Cancers (Basel). 2019 Aug 30;11(9):1276. doi: 10.3390/cancers11091276 (PMC6769894; doi:10.3390/cancers11091276)
Supplement: Supplementary file 1 [file cancers-11-01276-s001.pdf]

Supplementary Materials: Extracellular Vesicles from Human Advanced-Stage Prostate Cancer Cells Modify the Inflammatory Response of Microenvironment-Residing Cells

Letizia Mezzasoma, Egidia Costanzi, Paolo Scarpelli, Vincenzo Nicola Talesa and Ilaria Bellezza

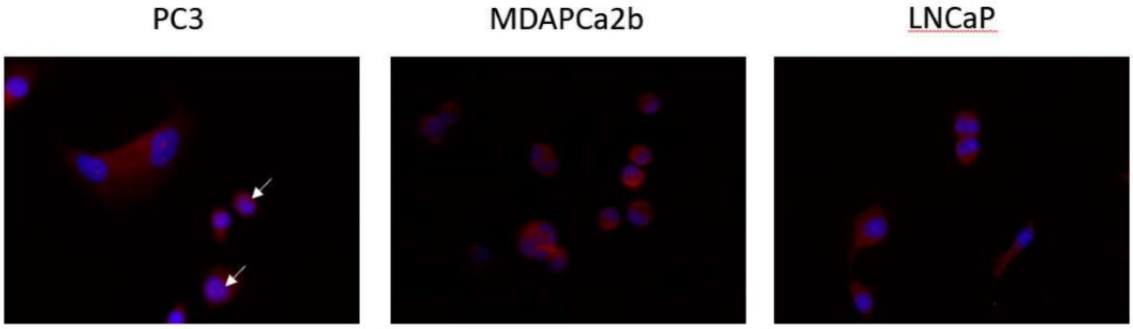

Figure S1. Nuclear localization of NF-κB. PC3, MDAPCa2B and LNCaP cells were fixed and used for the detection of NF-κB. Nuclei were counterstained with DAPI. The images are representative of one out of three separate experiments. Magnification 40 ×.

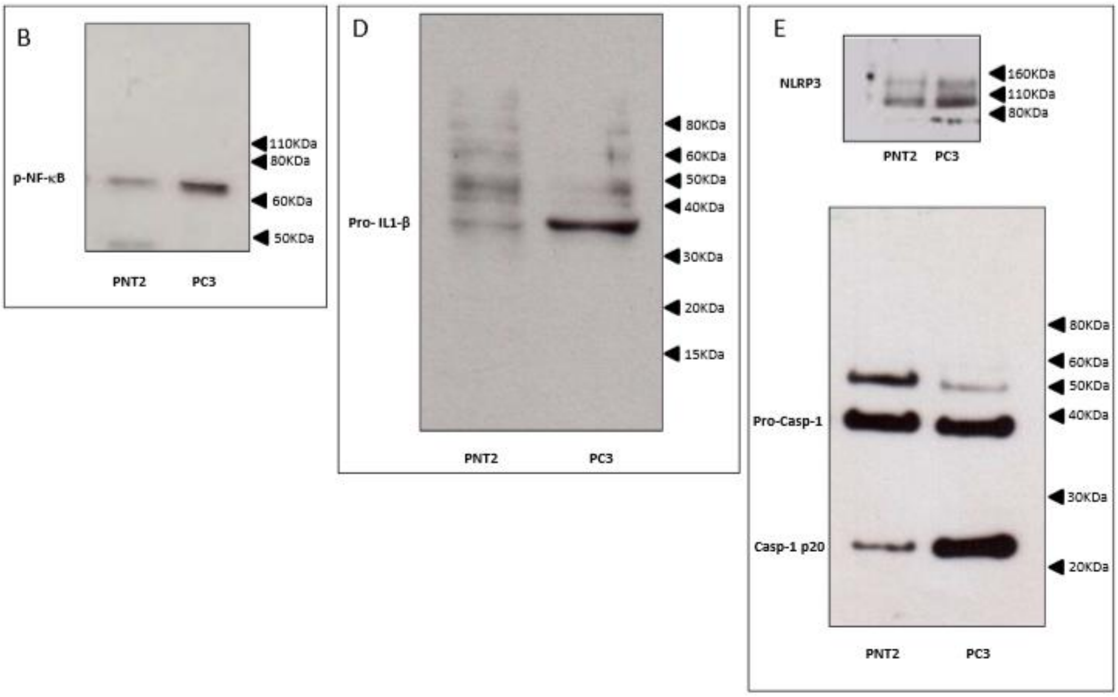

Figure S2. Whole blots reported in Figure.2.

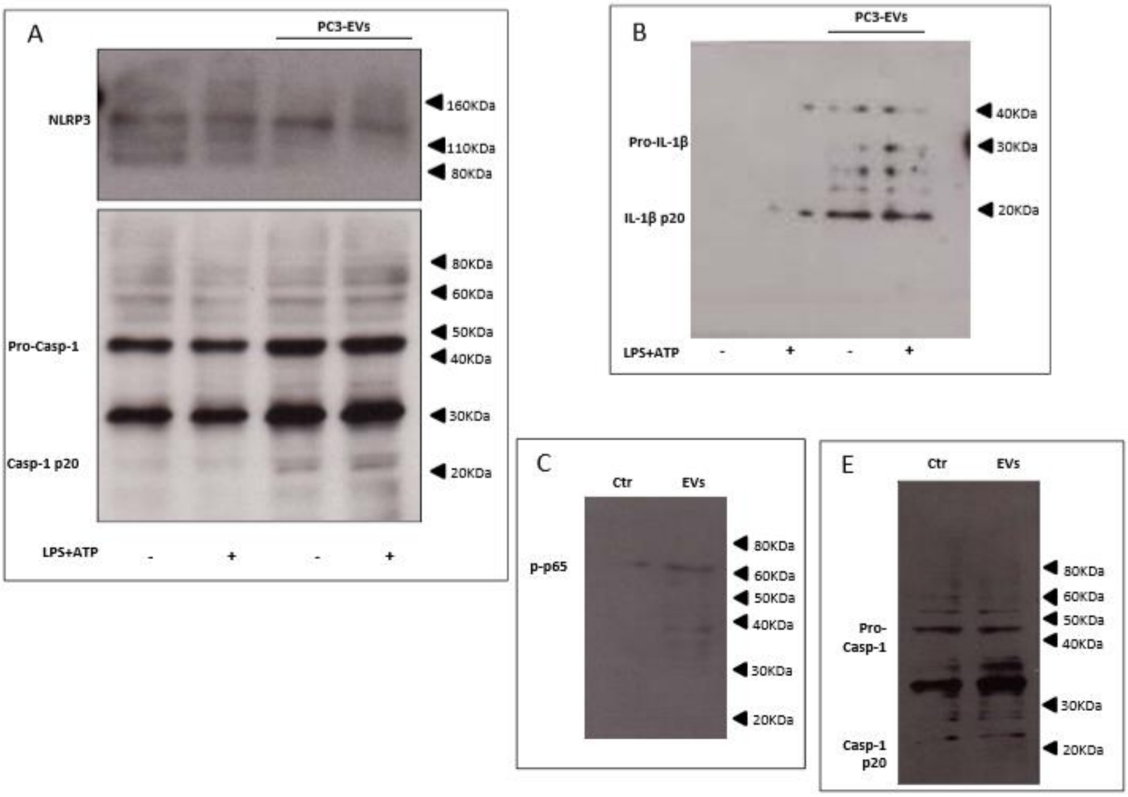

Figure S3. Whole blots reported in Figure.3.

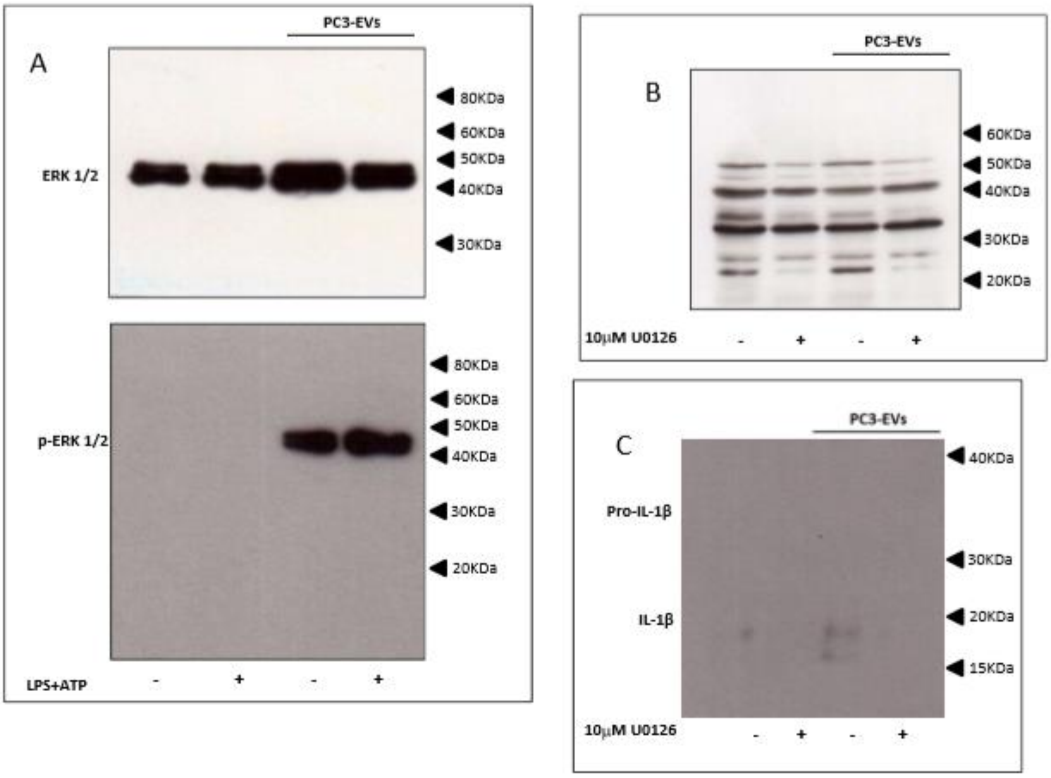

Figure S4. Whole blots reported in Figure.4.

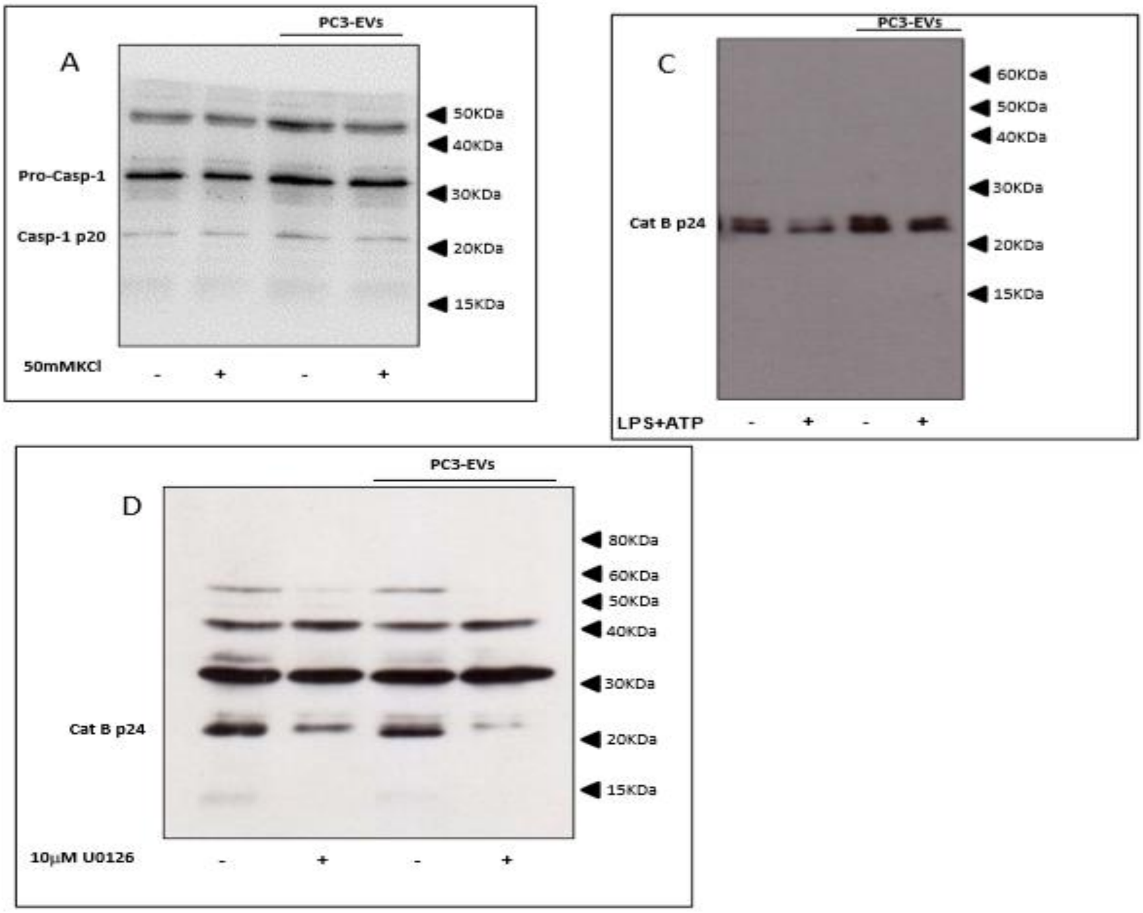

Figure S5. Whole blots reported in Figure.5.

© 2019 by the authors. Licensee MDPI, Basel, Switzerland. This article is an open access article distributed under the terms and conditions of the Creative Commons Attribution (CC BY) license (http://creativecommons.org/licenses/by/4.0/).
